# Comparison of microbiota structure in reproductive tract of Yanbian cattle and Yanhuang cattle

**DOI:** 10.3389/fmicb.2024.1419914

**Published:** 2024-07-31

**Authors:** Yunkun Teng, Shuai Feng, Zhuoxuan Gu, Chunqi Hou, Haoran Xu, Zhiqiang Li, Jing Zhao, Yi Fang, Xin Ma, Hongyu Liu, Jing Guo, Jun Wang, He Ding, Wenfa Lu

**Affiliations:** ^1^Key Lab of Animal Production, Product Quality and Security, Ministry of Education, Jilin Agricultural University, Changchun, China; ^2^Jilin Province Engineering Laboratory for Ruminant Reproductive Biotechnology and Healthy Production, College of Animal Science and Technology, Jilin Agricultural University, Changchun, China

**Keywords:** Yanbian cattle, Yanhuang cattle, microbiota, reproductive tract, 16S rRNA gene

## Abstract

Microbiota in the reproductive tract of cattle play a vital role in maintaining normal reproduction. However, the information on microbiota in different parts of reproductive tracts with different genetic background is few. The aim of the present study was to describe and compare the microbiota in vagina, cervix and uterus of Yanbian cattle and Yanhuang cattle. The results showed that microbial diversity increases from the vagina to the uterus. The top three bacterial phyla in bovine reproductive tract were *Proteobacteria*, *Firmicutes* and *Bacteroidetes*, accounting for more than 85%. From the vagina to the uterus, the relative abundance of *Proteobacteria* gradually decreased, while that of *Firmicutes* gradually increased. Phylum-level *Firmicutes* and genus-level *UCG_010* were significantly enriched in the uterus of Yanbian cattle and Yanhuang cattle. Comparing the same parts of the two breeds, it was found that there was no significant difference in alpha diversity, but significant differences in beta diversity. In addition, microbiota with significant differences in the relative abundance of the reproductive tract were found. These findings lay a foundation for a comprehensive understanding of the structure of the genital tract microbiota of cows and its regulatory mechanisms.

## Introduction

The genital tract microbiota plays an important role in cattle reproductive health. The reproductive tract microbiota can inhibit the invasion and proliferation of pathogens by forming biofilms (Tang et al., 2008; Tachedjian et al., 2017). Certain lactobacilli can protect fetal development during pregnancy and promote healthy delivery (Romero et al., 2014). Recent research shows that reproductive tract microbiota can transmit chemical signals between species by producing pheromones (Srinivasan et al., 2021). The dysbiosis leads to changes in the microbiota, including a decrease in the abundance of lactobacilli and an increase in the population of facultative anaerobes, leading to a predisposition of the host to a variety of diseases (Valenti et al., 2018; Saraf et al., 2021). Exploring the reproductive tract microbiota structure of healthy cattle will provide a solid theoretical basis for studying the occurrence of reproductive diseases and reproductive obstacles.

However, the microbes in the reproductive tract are not fixed. Bacteria can enter the vagina from the outside, including skin and feces, and transmitted to sites such as the cervix and uterus (Sheldon and Dobson, 2004). Bacteria can also enter the reproductive tract through the bloodstream route (Jeon et al., 2017). The vagina is still considered the main source of microbiota in the uterus, cervix and other parts of the body (Galvão et al., 2019). Pathogenic bacteria that cause uterine infections such as *Prevotella*, *Fusobacterium necrophorum*, *Escherichia coli*, *Arcanobacterium pyogenes*, etc. are often proven to be associated with the vagina (Deng et al., 2019). Therefore, the microbiota composition of the vagina, cervix, and uterus is expected to be closely related. Although previous studies have separately reported the structure of vaginal microbiota and uterine microbiota in cows (Vitale et al., 2021), and there is a lack of systematic exploration of the complete reproductive tract microbiota.

For studying the composition of microbiota in various parts of the cow’s reproductive tract, in addition to studying the correlation between the microbiota in various parts, its changes under breed factors should also be considered. Studies on Gyr (Giannattasio-Ferraz et al., 2019) and Nellore (Laguardia-Nascimento et al., 2015) cattle found significant differences in vaginal microbiota. However, previous studies on the impact of genetic factors on reproductive tract microbiota may be more affected by sampling region, season and nutritional factors, and there is a lack of research on the cervix and uterus. For this reason, it is necessary to eliminate interfering factors such as region, feeding management and feed differences that affect the reproductive tract microbiota as much as possible, and systematically study the impact of breed factors on the entire reproductive tract microbiota.

Yanbian cattle, one of the five major local fine-bred cattle in China, originated from 1850 to 1870 and was formed by cross-breeding Korean cattle and Mongolian cattle (Shen et al., 2020). Yanhuang cattle are made from Limousin cattle as the male parent and Yanbian cattle as the female parent, through cross-breeding, cross-fixation and group selection, this breed contains 75% of Yanbian cattle genes and 25% of Limousin cattle genes. Compared with Yanbian cattle, the growth and development of Yanhuang cattle at various stages has been significantly improved than that of Yanbian cattle. Yanhuang cattle have obvious advantages in slaughter performance and feed conversion ratio, and the digestibility of dietary nutrients is also higher than that of Yanbian cattle (Ji et al., 2014).

The aim of the study is to investigate the commonality and uniqueness of microorganisms in different parts of the reproductive tract of Yanbian cattle and Yanhuang cattle, as well as the influence of breed factors on the composition of microorganisms in the bovine reproductive tract, which laid a foundation for a comprehensive understanding of the microecological composition and regulation of bovine genital tract.

## Methods

### Animals and samples

This study selected Yanbian cattle and Yanhuang cattle from Benfu Ranch in Yanbian Korean Autonomous Prefecture, China. Multiparous cattle with healthy body condition and aged 3–5 years were selected. Samples were collected from November and December 2022. In order to avoid cows in different physiological cycles affecting the structure of the reproductive tract microbiota, we use estrus identification technology to select cows that were naturally in estrus (external observation combined with vaginal examination). Cotton swabs was used to collect samples on the day of estrus, and samples from the reproductive tract including the vagina, cervix and uterus were collected. A total of 98 samples from 21 Yanbian cattle (vagina: *n* = 14; cervix: *n* = 17; uterus: *n* = 17) and 19 Yanhuang cattle (vagina: *n* = 17; cervix: *n* = 17; uterus: *n* = 16) were collected for subsequent sequencing analysis. Samples were collected on sterile cotton swabs, placed in cryovials, group the samples (A.V: Yanbian cattle vagina; A.C: Yanbian cattle cervix; A.U: Yanbian cattle uterus; B.V: Yanhuang cattle vagina; B.C: Yanhuang cattle cervix; B.U: Yanhuang cattle uterus), and stored in liquid nitrogen at −196°C until used for microbiome analysis.

### DNA extraction and sequencing

Genomic DNA was extracted by CTAB method, and DNA purity and concentration were detected using 1% agarose gel electrophoresis. Take an appropriate amount of sample DNA in a centrifuge tube and use sterile water to dilute the sample to 1 ng/μL. PCR amplification of the bacterial 16S rRNA gene V3–V4 region was performed using the forward primer 341F (5′-CCTAYGGGRBGCASCAG -3′) and the reverse primer 806R (5′-GGACTACNNGGGTATCTAAT-3′) (Table 1). Add 15 μL Phusion^®^ High-Fidelity PCR Master Mix (New England Biolabs), 0.2 μM primer and 10 ng genomic DNA template to the PCR mixture, perform the first denaturation at 98°C for 1 min, and then denature at 98°C (10 s) for 50°C (30 s) and 72°C (30 s) for 30 cycles, and it was maintained at 72°C for 5 min to obtain the PCR product. The expected amplified product fragment was 470 bp. PCR products are detected by electrophoresis using 2% concentration agarose gel; qualified PCR products are purified with magnetic beads and quantified using enzyme labeling. Equal amounts of samples are mixed according to the concentration of the PCR product. After mixing thoroughly, use 2% agarose gel electrophoresis to detect the PCR product, and use a universal DNA purification and recovery kit (TianGen) to recover the product of the target band. NEB Next^®^ Ultra^™^ II FS DNA PCR-free Library Prep Kit (New England Biolabs) was used for library construction. The constructed library was quantified by Qubit and Q-PCR. After the library was qualified, NovaSeq 6000 (518 cycles) was used for PE 250 On-machine sequencing. All sequences used in this study are publicly available at the NCBI Sequence Read Archive under accession ID PRJNA1129596.

**Table 1:** Primers sequences for PCR.

Region	Primer name	Primer sequence (5′–3′)	Product (bp)
Bacteria 16S	V3 + V4	341F	CCTAYGGGRBGCASCAG
		806R	GGACTACNNGGGTATCTAAT

### Sequence analysis

Illumina NovaSeq sequencing platform was used for double-terminal sequencing of the library. Briefly, according to the Barcode sequence and PCR amplification primer sequence, each sample data is split from the offline data. Double-end data splicing uses FLASH (Version 1.2.11, http://ccb.jhu.edu/software/FLASH/) to truncate the Barcode and primer sequences to splice the reads of each sample, and the resulting spliced sequence is: Raw Tags data (Raw Tags). Use fastp software (Version 0.23.1) to perform strict filtering on the spliced Raw Tags to obtain high-quality Tags data (Clean Tags). The Tags obtained after the above processing need to be processed to remove chimera sequences. The Tags sequences are compared with the species annotation database (Silva database https://www.arb-silva.de/ for16S) to perform comparison and detection of chimeric sequences, and finally remove the chimeric sequences to obtain the final effective data (Effective Tags). For the obtained EffectiveTags, the DADA2 module or deblur in the QIIME2 (VersionQIIME2-202006) software was used for denoising (DADA2) to obtain the final ASVs (Amplicon Sequence Variants) and feature tables. Classify-sklearn algorithm of QIIME2 is adopted (Bokulich et al., 2018; Bolyen et al., 2019) annotated species for each ASV using a pre-trained Naive Bayes classifier.

### Bioinformatic analysis

Sequence data analysis was mainly performed using the QIIME2 and R packages. According to the results of ASVs annotation and the characteristic table of each sample, the top 10 dominant species were screened from the species abundance table at phylum and genus level, and the relative species abundance histogram was generated. A Venn diagram was generated to visualize the shared and unique OTUs among samples or groups using the R (Version 3.5.3) package “Venn Diagram.” Use QIIME2 software to calculate α diversity indices such as Chao1 index and Shannon index. β-diversity analysis was performed using the Bray–Curtis index to investigate structural changes in microbial communities in the sample and visualized by generating PCoA distribution maps. LDA effect size (LDA score >4) was performed to detect differentially abundant taxa across groups using the default parameters. In R (Version 3.5.3), a *t*-test was used to compare the abundance of taxa at the phyla and genus level within the group.

## Results

### Comparison of vaginal, cervical and uterine microbiota

**Figure 1:**
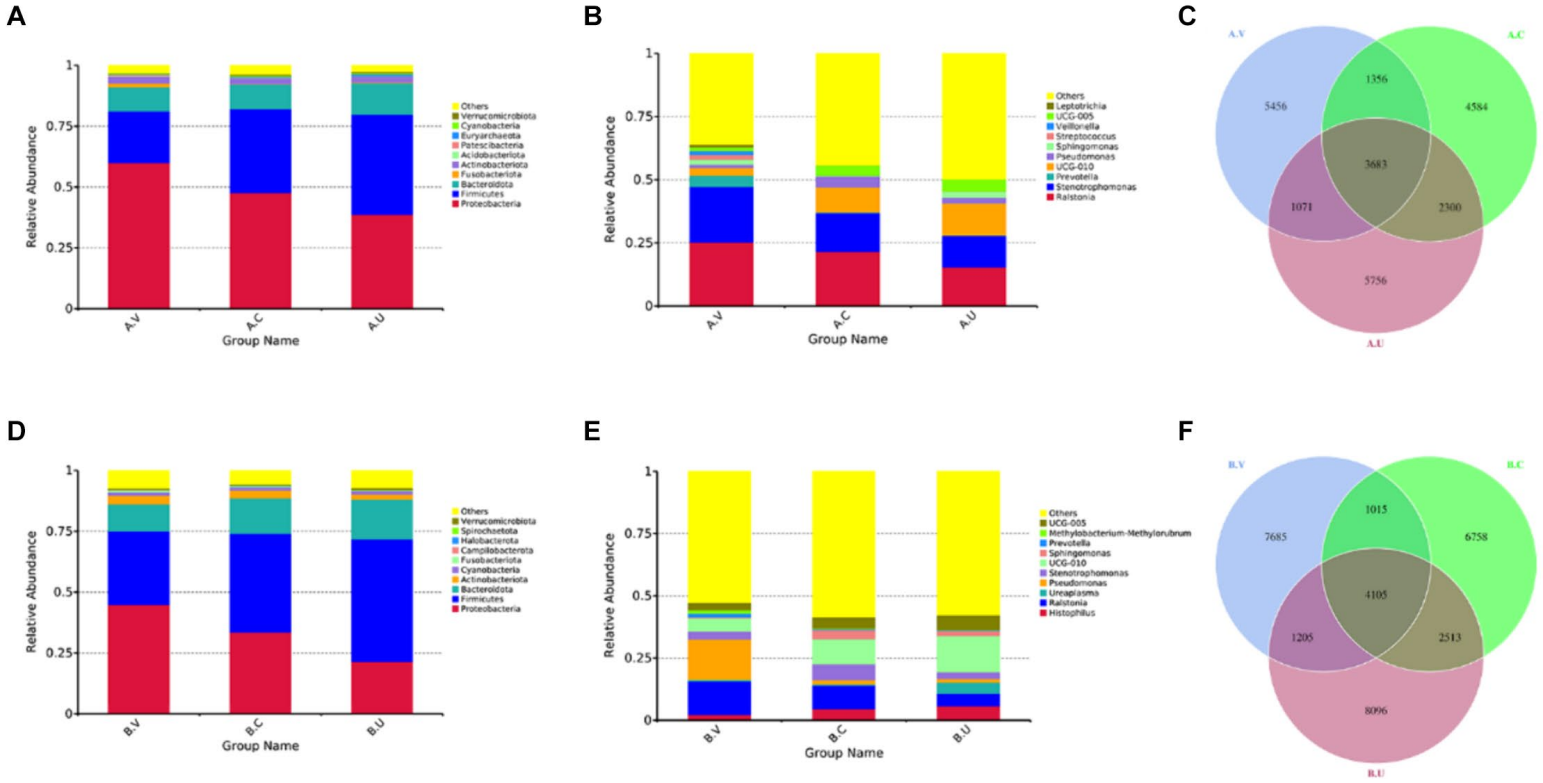
Composition and proportion of vaginal, uterine and cervical microbiota. **(A,B,D,E)** are the species annotations and abundance information at the phylum and genus level of the vagina, cervix, and uterus of Yanbian cattle and Yanhuang cattle. The top 10 species with the highest abundance were selected to generate a column accumulation chart of species relative abundance, the abscissa is the group name; the ordinate represents the relative abundance; others represents the sum of the relative abundance of all other phyla except these 10 phyla in the figure. Each circle in the figures **C,F** represents a group. The number in the overlapping part of the circle and the circle represents the number of OTUs shared between the groups. The number in the non-overlapping part represents the number of unique OTUs of the group.

Through the species relative abundance column chart, we can visually see the species and their proportions with higher relative abundance in each group at the phylum and genus level. As shown in Figure 1A, the top three dominant bacterial phyla in the reproductive tract of Yanbian cattle are *Proteobacteria* (uterus: 38.63%, cervix: 47.65%, vagina 59.97%), *Firmicutes* (uterus: 41.19%, cervix: 34.56%, vagina: 21.27%), *Bacteroidota* (uterus: 12.90%, cervix: 10.11%, vagina: 9.97%). The proportion of the top three dominant bacterial phyla in various reproductive tract parts of Yanbian cattle is as high as 91.35–92.72%. The top three bacterial phyla in the reproductive tract of Yanhuang cattle are *Proteobacteria* (uterus: 21.37%, cervix: 33.60%, vagina: 30.30%), *Firmicutes* (uterus: 50.48%, cervix: 40.50%, vagina: 30.30%), *Bacteroidota* (uterus: 16.25%, cervix: 14.58%, vagina: 11.12%) (Figure 1D). The proportion of the top three dominant bacterial phyla in various reproductive tract parts of Yanhuang cattle is as high as 86.18–88.68%. The results showed that the top three dominant phyla of the two breeds of cattle were *Proteobacteria*, *Firmicutes* and *Bacteroidetes*, and these three phyla showed a consistent trend in the reproductive tract: the relative abundance of *Proteobacteria* showed a gradient decreasing trend from the vagina to the uterus, and the relative abundance of *Firmicutes* showed a gradient increasing trend from the vagina to the uterus, there was no obvious change in the *Bacteroidetes* phylum. From the genus level, *Ralstonia* occupies a high abundance in the reproductive tract of Yanbian cattle and Yanhuang cattle, and the relative abundance gradually decreases from the vagina to the uterus. As another dominant genus, *UCG-010* has a relative abundance that gradually increases from the vagina to the uterus of Yanbian cattle and Yanhuang cattle. The *Histophilus* only occupies a greater advantage in the reproductive tract of Yanhuang cattle, and the relative abundance of *Histophilus* showed an increasing trend from the vagina to the uterus of Yanhuang cattle (Figures 1B,E).

As shown in Figures 1C,F, the number of OTUs that coexisted in the vagina, cervix and uterus of Yanbian cattle reached 3,683, 5,039 OTUs coexisted in the vagina and cervix, 5,983 OTUs coexisted in the cervix and uterus, and 4,754 OTUs coexisted in the vagina and uterus. The number of OTUs that coexisted in the vagina, cervix and uterus of Yanhuang cattle reached 4,105, 5,120 OTUs coexisted in the vagina and cervix, 6,618 OTUs coexisted in the cervix and uterus, and 5,310 OTUs coexisted in the vagina and uterus. In addition, there are still relatively high amounts of OTU in the vagina, cervix, and uterus of the two breeds of cattle, which are unique to each part.

**Figure 2:**
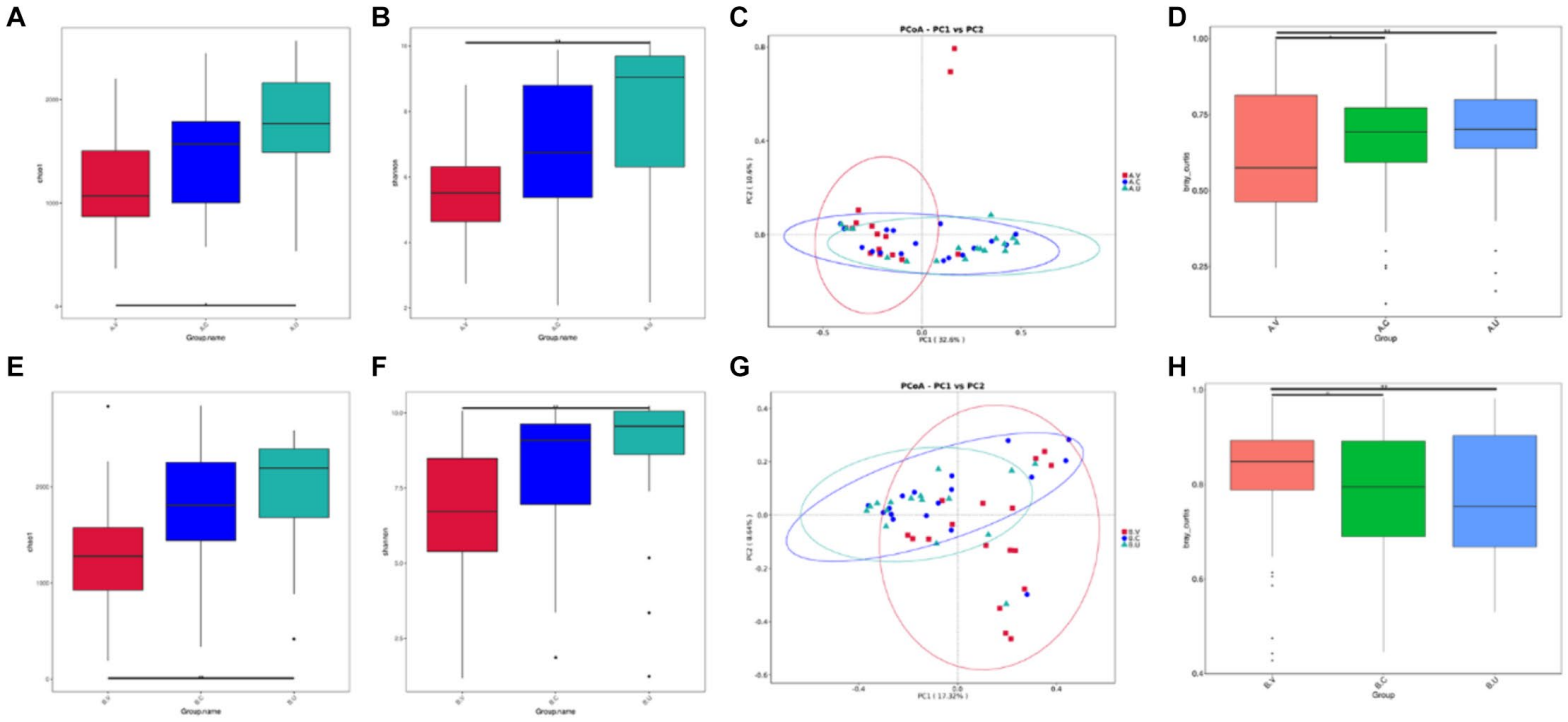
Comparison of the alpha and beta diversity of the vaginal, cervical and intrauterine microbiota of Yanbian cattle and Yanhuang cattle. Alpha diversity, including Chao 1 and Shannon index **(A,B,E,F)** of the two groups of samples, **C,G** based on the beta diversity of the Bray–Curtis metric of Yanbian cattle and Yanhuang cattle. The abscissa represents one principal component, the ordinate represents another principal component, and the percentage represents the contribution of the principal component to the sample difference; each point in the figure represents a sample, and samples in the same group are represented by the same color. **(D,H)** The Wilcox rank sum test was used to analyze the Bray–Curtis differences among each group. ^*^Means significant difference (*p* < 0.05), ^**^means extremely significant difference (*p* < 0.01).

By comparing the differences in Shannon index and Chao 1 index of the vagina, cervix and uterus of Yanbian cattle and Yanhuang cattle (Figures 2A,B,E,F), it was found that the alpha diversity changes of the reproductive tract microbiota of Yanbian cattle and Yanhuang cattle showed consistent changes. There was a significant difference in the alpha diversity of the vagina and uterus between the two breeds (*p* < 0.05). However, there was no significant difference in alpha diversity between vagina-cervix and cervix-uterus (*p* > 0.05). Based on the PCoA distribution of vaginal, cervical and uterine samples from the two breeds of cattle, it was found that the distribution of vaginal samples was significantly different from the distribution of uterine and cervical samples, while the distribution of cervical and uterine samples was more similar and the community structure was more similar (Figures 2C,G). In order to further determine the significance of distribution differences between sample groups, using the Wilcox rank sum test method based on Bray–Curtis, there were significant differences in vaginal-cervical and vaginal-uterine beta diversity (*p* < 0.05), and there was no significant difference in cervical-uterine beta diversity (*p* > 0.05) (Figures 2D,H).

**Figure 3:**
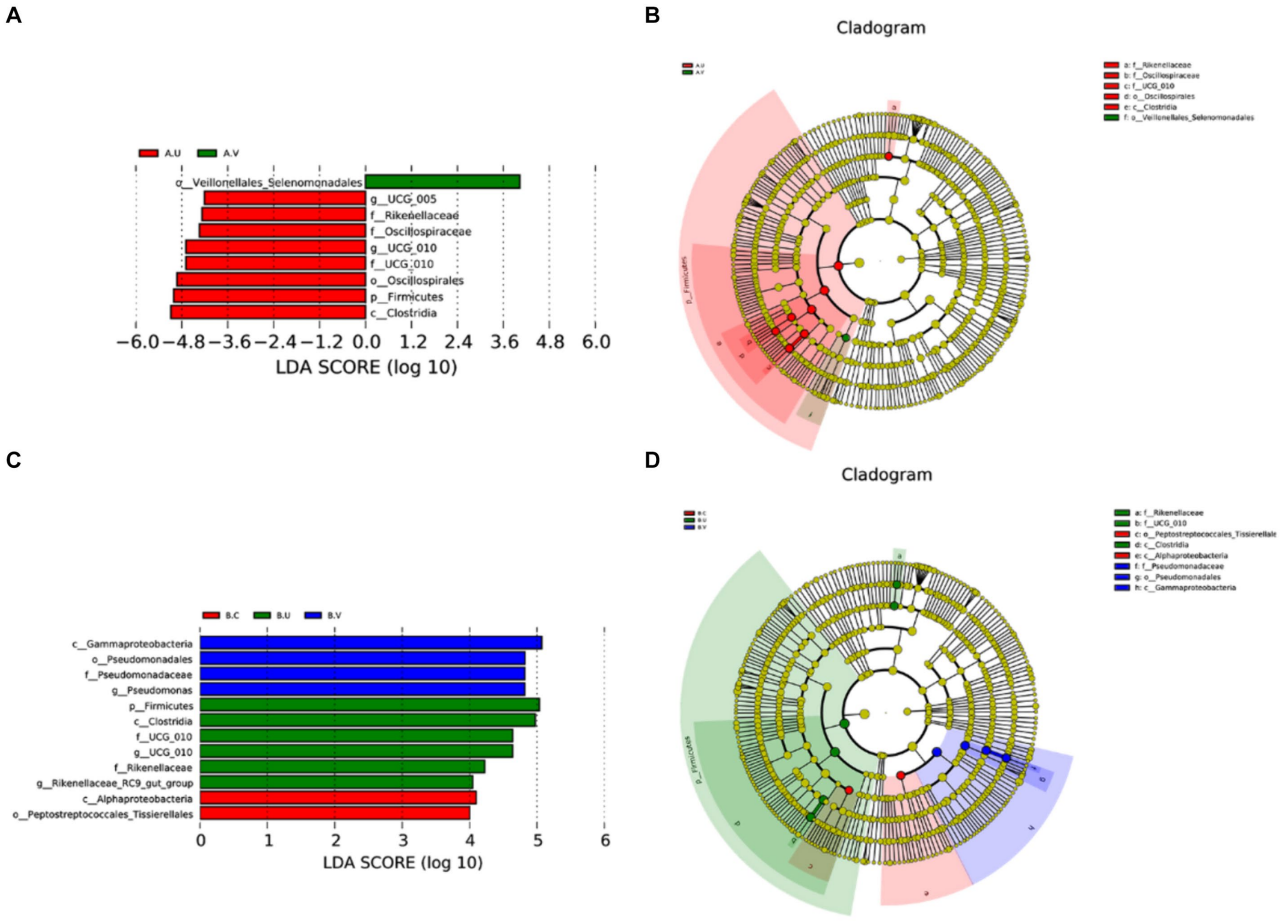
Comparison of the microbiota in the vagina, cervix and uterus of Yanbian cattle and Yanhuang cattle. The LDA value distribution histogram shows species whose LDA score is greater than the set value of 4, that is, species that are significantly enriched in each group. The length of the bar graph represents the effect size (LDA score) of differential species **(A,C)**. In a cladogram, the circles radiating from the inside to the outside represent the classification levels from phylum to genus (or species). Each small circle at a different classification level represents a classification at that level, and the diameter of the small circle is proportional to the relative abundance **(B,D)**.

In order to find out the species with significant differences in different genital tract parts between Yanbian cattle and Yanhuang cattle, statistics of significantly enriched bacterial groups in each group were carried out through LEfSe analysis. As shown in Figure 3. *o_Veillonellales_Selenomonadales* was significantly enriched in the vagina of Yanbian cattle, and *c_Gammaproteobacteria*, *o_Pseudomonadales*, and *f_Pseudomonadaceae* were significantly enriched in the vagina of Yanhuang cattle. In the uterus of Yanbian cattle and Yanhuang cattle, *f_Rikenellaceae*, *g_UCG_010*, *f_UCG_010*, *p_Firmicutes*, and *c_Clostridia* were significantly enriched. *g_UCG-005*, *f_Oscillospiraceae*, and *o_Oscillospirales* were only enriched in the uterus of Yanbian cattle. *g_Rikenellaceae_RC9_gut_group* was only significantly enriched in the uterus of Yanhuang cattle. Thus, phylum-level *Firmicutes* and genus-level *UCG_010* were significantly enriched in the uterus of two breeds of cattle, *UCG-005* was significantly enriched only in the uterus of Yanbian cattle, and *Rikenellaceae_RC9_gut_group* was significantly enriched only in the uterus of Yanhuang cattle (Figures 3A,C).

### Comparison of microbiota in the same reproductive tract between Yanbian cattle and Yanhuang cattle

**Figure 4:**
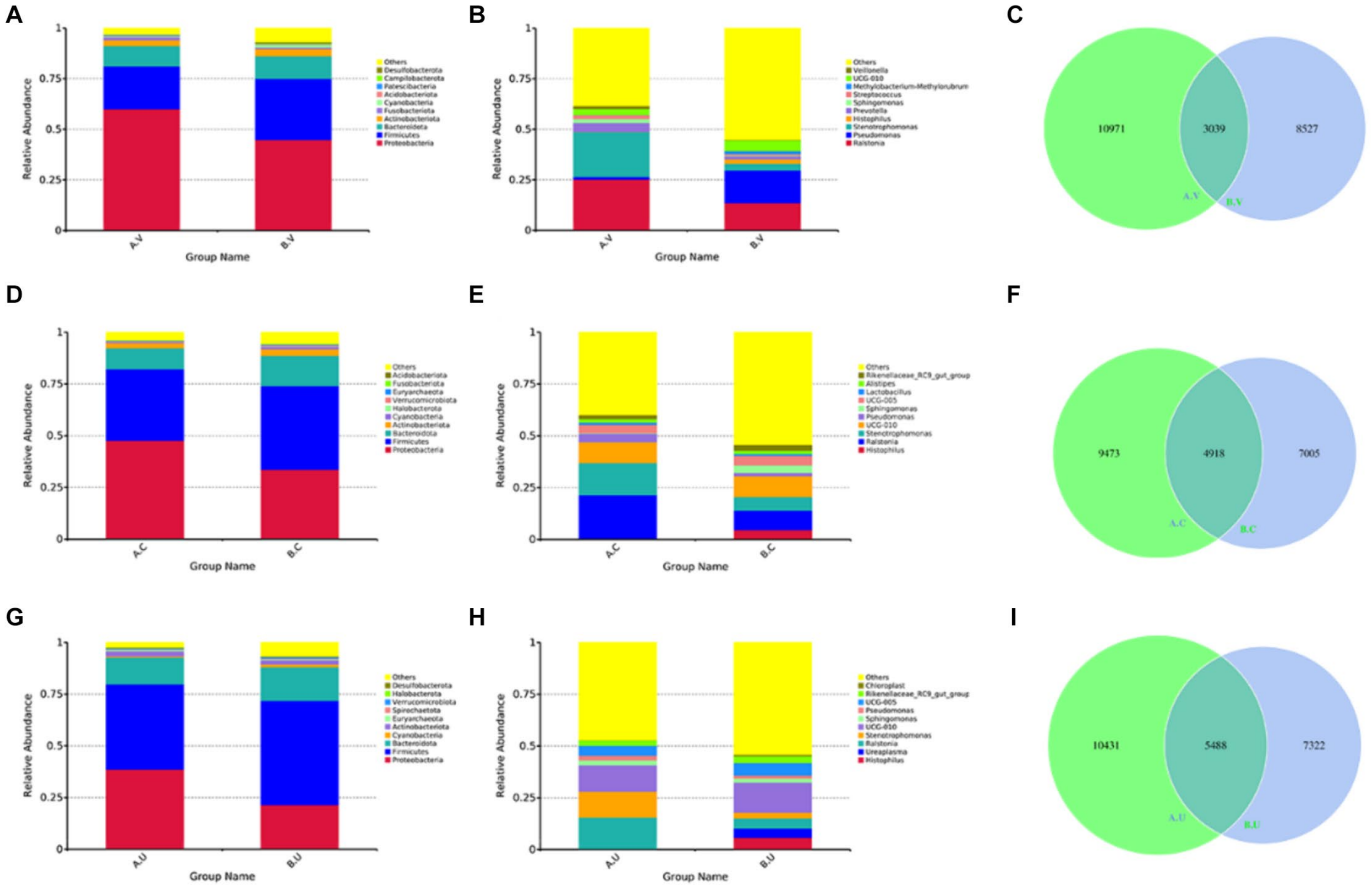
The composition and proportion of the microbiota in the same reproductive tract of Yanbian cattle and Yanhuang cattle. **(A,B,D,E,G,H)** Are the species annotations and abundance information at the phylum and genus level in the same reproductive tract of Yanbian cattle and Yanhuang cattle. Select the top 10 species with the highest abundance to generate a species relative abundance column. It is a cumulative graph. The abscissa (group name) is the group name; the ordinate (relative abundance) represents the relative abundance; others represents the sum of the relative abundance of all other phyla except the 10 phyla in the graph. Each circle in the figures **C,F,I** represent a group. The number in the overlapping part of the circle and the circle represents the number of OTUs shared between the groups. The number in the non-overlapping part represents the number of unique OTUs of the group.

In order to study the main composition and proportion of microbiota in the same reproductive tract of Yanbian cattle and Yanhuang cattle, the top 10 species with the highest abundance were selected to generate a column accumulation chart of species relative abundance. In order to visually observe the distribution of dominant species among groups. The results showed that at the phylum level, the top three bacterial phyla were *Proteobacteria*, *Firmicutes*, and *Bacteroidetes*. Among them, the relative abundance of *Proteobacteria* in the vagina, cervix and uterus of Yanbian cattle was higher than that of Yanhuang cattle, while the relative abundance of *Firmicutes* and *Bacteroidetes* was lower than that of Yanhuang cattle (Figures 4A,D,G). In addition, at the genus level, *Ralstonia* and *Stenotrophomonas* were both higher in the vagina, cervix and uterus of Yanbian cattle to a certain extent than in Yanhuang cattle (Figures 4B,E,H). Interestingly, the cervix and uterus of Yanhuang cattle also contain a relatively high abundance of *Histophilus*. The OTU of the vagina, cervix and uterus of the two breeds of cattle showed that the number of OTU from the vagina to the uterus of the two breeds of cattle increased (vagina: 3039 OTUs, cervix: 4918 OTUs, uterus: 5488 OTUs) (Figures 4C,F,I).

**Figure 5:**
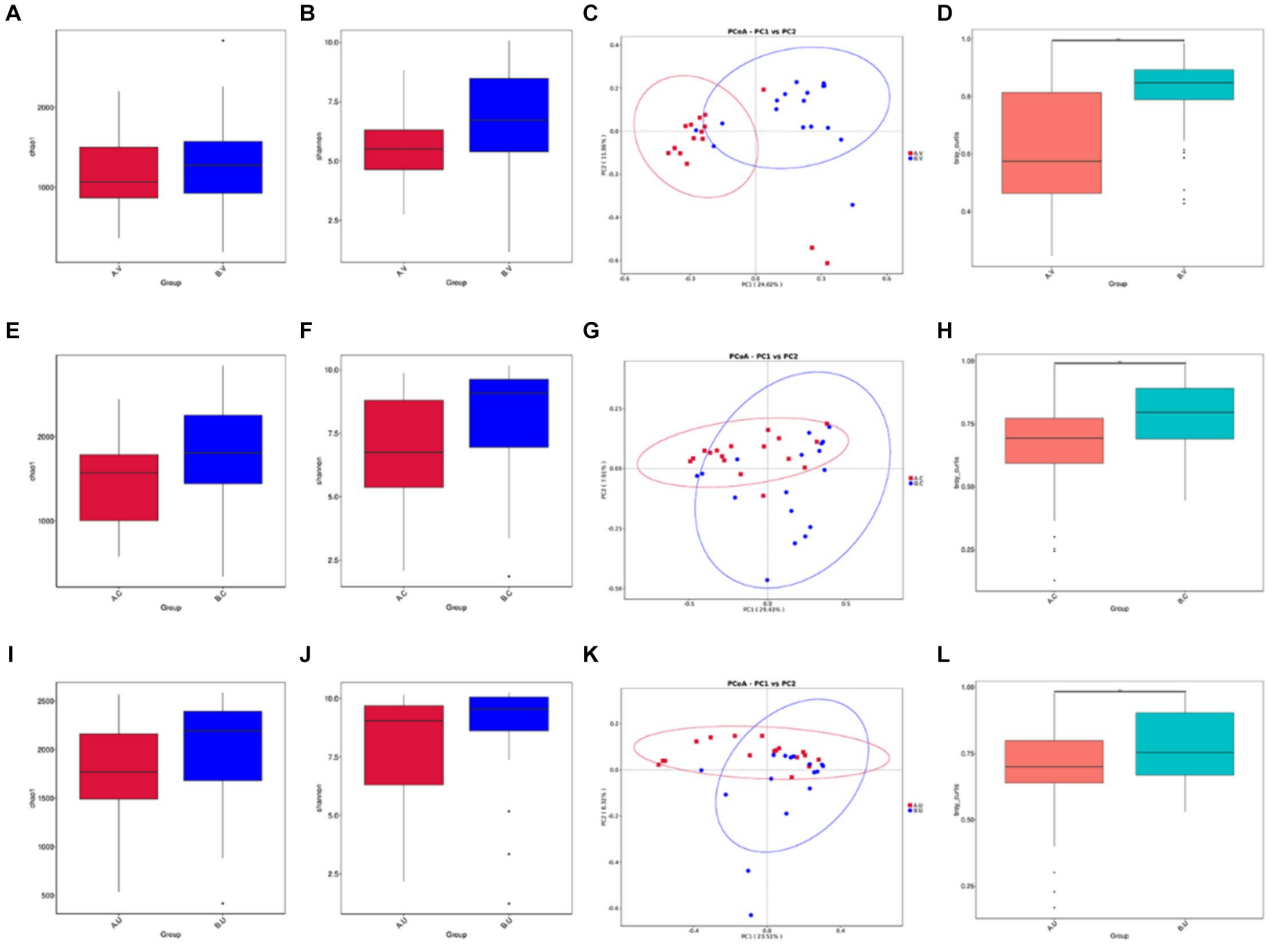
Comparison of the alpha and beta diversity of the microbiota in the same reproductive tract site of Yanbian cattle and Yanhuang cattle. Alpha diversity, including Chao 1 and Shannon index **(A,B,E,F,I,J)** of the two groups of samples, **C,G,K** based on the beta diversity of the Bray–Curtis metric of Yanbian cattle and Yanhuang cattle. The abscissa represents one principal component, the ordinate represents another principal component, and the percentage represents the contribution of the principal component to the sample difference; each point in the figure represents a sample, and samples in the same group are represented by the same color. **(D,H,L)** The Wilcox rank sum test was used to analyze the Bray–Curtis differences among each group. ^*^Means significant difference (*p* < 0.05).

In order to further explore the influence of genetic factors on the diversity of reproductive tract microbiota, we compared the diversity of the same genital tract microbiota between Yanbian cattle and Yanhuang cattle. Regarding alpha diversity, as shown by the Chao 1 index (Figures 5A,E,I) and Shannon index (Figures 5B,F,J), the richness and evenness of the reproductive tract microbiota of Yanhuang cattle are slightly higher than those of Yanbian cattle, but there is no significant difference (*p* > 0.05). We analyzed beta diversity using the Bray–Curtis based Wilcox rank sum test method to examine differences in microbial communities between groups, there is a large dispersion in the distribution of vaginal, cervix, and uterine samples (Figures 5C,G,K), and the differences in the microbial communities in the same parts were statistically analyzed. As shown in Figures 5D,H,L, there were significant differences in beta diversity between the vagina, cervix, and uterus of Yanbian cattle and Yanhuang cattle.

**Figure 6:**
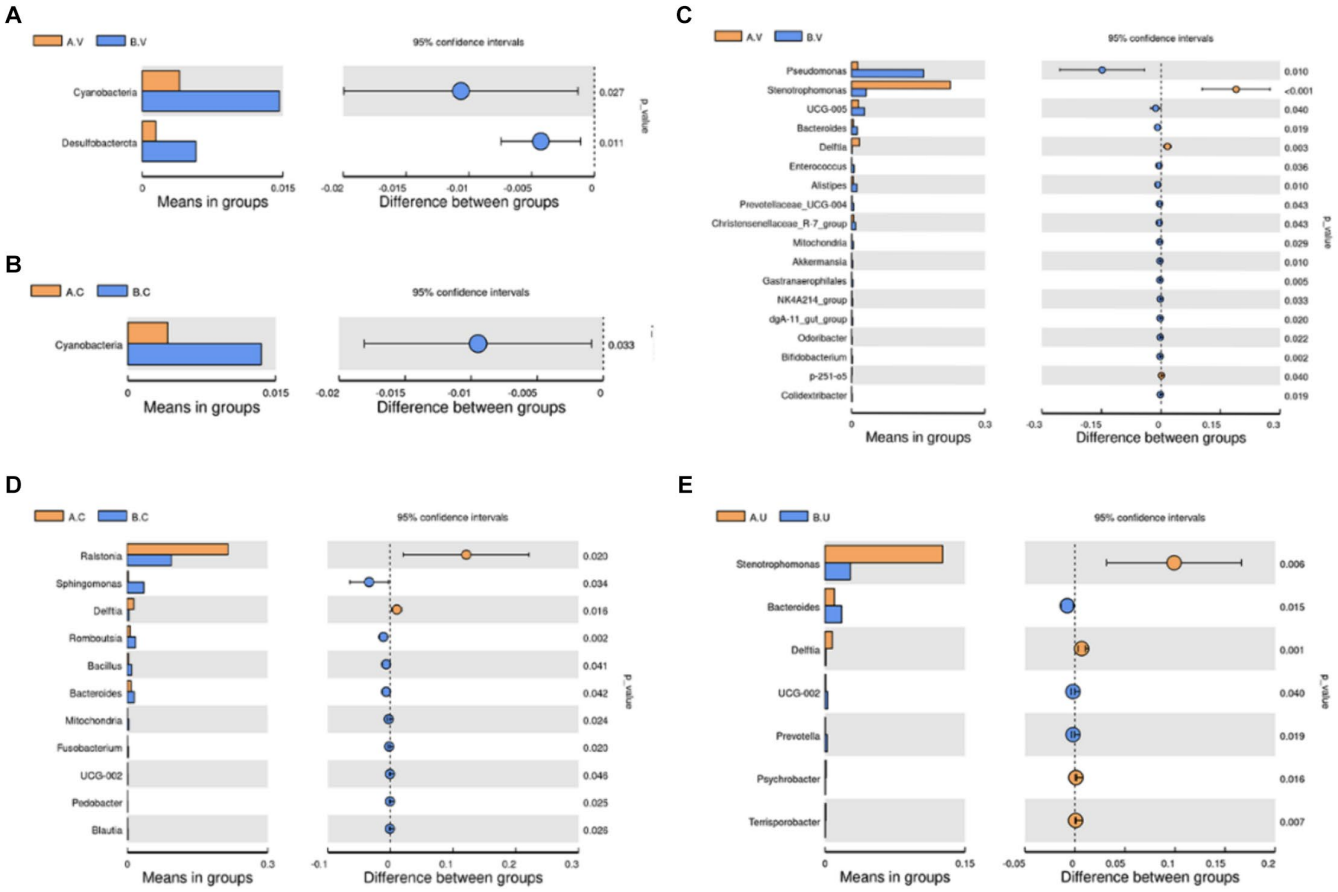
Comparison of the microbiota in the same reproductive tract of Yanbian cattle and Yanhuang cattle. Use *t*-test to identify species with significant differences between groups at the phylum **(A,B)** and genus **(C–E)** levels (*p* < 0.05). The picture on the left shows the difference in species abundance between groups. Each bar in the picture represents the mean value in each group of species with significant differences in abundance between groups. The picture on the right shows the confidence level of the difference between groups. The leftmost endpoint of each circle in the picture represents the lower limit of the 95% confidence interval of the mean difference, and the rightmost endpoint of the circle represents the upper limit of the 95% confidence interval of the mean difference. The center of the circle represents the difference between the means, and the color of the circle represents the *p*-value of the significant difference test between groups for the corresponding species.

In order to further identify the differential species that affect the bacterial community structure, *t*-test was used to identify species with significant differences at the phylum and genus levels, and then clarify the next research direction. At the phylum level, the number of *Cyanobacteria* in the vagina and cervix of Yanbian cattle was significantly lower than that of Yanhuang cattle (*p* < 0.05), and the number of *Desulfobacterota* in the vagina of Yanbian cattle was significantly lower than that of Yanhuang cattle (Figures 6A,B). No phylum-level species differences were observed in the uterus of Yanbian cattle and Yanhuang cattle. At the genus level, *Delftia* was significantly higher in the vagina, cervix, and uterus of Yanbian cattle than in Yanhuang cattle; *Stenobacteria* was significantly higher in the vagina, uterus of Yanbian cattle. In Yanbian cattle. In addition, *Bacteroides* was significantly lower in the vagina, cervix, and uterus of Yanbian cattle than in Yanhuang cattle, and *Mitochondria* was significantly lower in the vagina and cervix of Yanbian cattle than in Yanhuang cattle (Figures 6C–E).

## Discussion

The results of this study show that the abundance and composition of bacteria in the vagina, cervix and uterus are common and different. In addition, there were differences in the composition of microbiota in the same part between the two breeds. In this study, the top three bacterial phyla in the vagina-cervix-uterus are *Proteobacteria*, *Firmicutes*, and *Bacteroidetes*. From the vagina to the uterus, the relative abundance of *Proteobacteria* shows a gradient decreasing trend. From the vagina to the uterus, the relative abundance of *Muricobacteria* showed a gradient increasing trend, and Yanbian cattle and Yanhuang cattle showed a consistent pattern. These three bacterial phyla dominate the digestive tract and may be related to the main source of reproductive tract microbiota (Zhu et al., 2023). Previous studies on the reproductive tract microbiota of humans (Liu et al., 2022) and cattle (Clemmons et al., 2017) have proven the dominance of these three bacterial phyla, but the proportions of each bacterial phylum are different, which may be related to environmental, nutritional and other factors. In the first three bacterial phyla, *Proteobacteria* include most of the well-known pathogenic bacteria and are potential diagnostic features of dysbiosis or disease risk (Chen et al., 2021). Studies have shown that *Firmicutes* in the digestive tract are mostly obligate anaerobic bacteria (Wozniak et al., 2022), this may be the reason for the presence of high-abundance *Firmicutes* in the anaerobic environment of the uterus. Studies have shown that the ratio of *Firmicutes* to *Bacteroidetes* is related to the occurrence of inflammation (Wu et al., 2021). Artificial control of the ratio of the bovine reproductive tract microbiota, such as the infusion of probiotics, has important potential in preventing inflammatory diseases in the bovine reproductive tract. Interestingly, we found that *Histophilus* was enriched in the reproductive tract of Yanhuang cattle, and the relative abundance increased from the vagina to the uterus. *Histophilus* is associated with several disease syndromes in cattle (Shirbroun, 2020). More recent studies indicate that *Histophilus* stimulates endothelial cell tissue factor activity and disrupts intercellular junctions (Behling-Kelly et al., 2016).

The results of the study showed an increasing trend in α diversity from the vagina to the uterus, and there are significant differences in alpha diversity between vagina and uterus, which is consistent with previous studies on human reproductive tract microbiota (Chen et al., 2017; Liu et al., 2022). However, previous studies of lactating Angus cattle have found that the alpha diversity of the vaginal flora is significantly higher than that of the uterine flora (Clemmons et al., 2017). Compared with our study, there are differences in season, region, breed, sampling method and animal feeding management mode, etc. These factors may lead to differences in the results of the two studies. From the perspective of physiological structure, the environment of tight cervix may also be an important reason for the difference in diversity of vaginal and uterine microbiota. As one of the important barriers to protect the uterine body from environmental pathogens, the cervix maintains a more stable environment of the uterus (Sheldon and Dobson, 2004; Azawi, 2008). The anaerobic environment *in utero* has a low biomass of bacteria, but corresponds to a high bacterial diversity (Chen et al., 2017). Besides, there are significant differences in beta diversity between vagina-cervix and vagina-uterus. This highlights the fact that there are differences in vaginal-cervical and vaginal-uterine microbiota profiles. *Firmicutes* were significantly enriched in the uterus of two breeds of cattle, although previous studies revealed that *Firmicutes* occupies a dominant position in the uterus (Machado et al., 2012; Santos and Bicalho, 2012; Clemmons et al., 2017), previous studies have also revealed the dominant position of *Firmicutes* in uterus. In addition, *UCG-010* was significantly enriched in the uterus of two breeds of cattle. Previous studies have demonstrated the enrichment of *UCG-010* in the intestine of cattle (Couch et al., 2021). The presence of this bacteria in the uterus may originate from the digestive tract. At the same time, the uterine environment may be more conducive to the colonization of *UCG-010*, but its role in the uterus has not yet been reported. However, *UCG-005* was only significantly enriched in the uterus of Yanbian cattle, and *Rikenellaceae_RC9_gut_group* was only significantly enriched in the uterus of Yanhuang cattle. Studies have shown that members of *Rikenellaceae_RC9_gut_group* can neutralize cytotoxic reactive oxygen species and protect cells from oxidative stress, thereby reducing the likelihood of inflammation (Gryaznova et al., 2022). We found that Yanhuang cattle only had common marker species in the uterus, and the number of marker species showed an increasing trend from vagina to uterus. This finding suggests that the uterus may have a more stable environment, making the microbiota more similar in the uterus of different species. In addition to being affected by genetic factors between breeds, the vagina and cervix are more affected by opportunistic infections in the external environment.

The research on the factor of variety shows that the relative abundance of *Proteobacteria* in the vagina, cervix and uterus of Yanbian cattle is higher than that of Yanhuang cattle, and the relative abundance of *Firmicutes* and *Bacteroidetes* is lower than that of Yanhuang cattle. The number of OTU in the uterus of Yanbian cattle and Yanhuang cattle was the highest. In previous studies, a total of 2075 OTUs were found in the vaginas of Holstein and Fleckvieh cattle (Nesengani et al., 2017), close to the results of this study. This further proves that there is a more stable environment in the uterus, making the uterus of different breeds of cattle have more similar microbiota. Previous studies have also revealed high similarities in the uterine microbiota of dairy cows in the same state (Santos et al., 2011). There is no significant difference in the alpha diversity of the vagina, cervix and uterus of the two breeds of cattle, but the alpha diversity of the microbiota in various parts of the reproductive tract of Yanhuang cattle is slightly higher than that of Yanbian cattle. The differences in species composition between different habitats were analyzed through beta diversity. There are significant differences in the characteristics of the microbiota in the same reproductive tract of cattle breeds. This diversity difference can be explained by different breeds (Appiah et al., 2020). To this end, we further identified the species responsible for this difference at the phylum and genus level. We found that the number of *Cyanobacteria* in the vagina and cervix of Yanbian cattle was significantly lower than that of Yanhuang cattle, *Cyanobacteria* are also highly enriched in the intestines of other animals, such as yaks (Wang et al., 2021), but there is currently no scientific evidence that *Cyanobacteria* play a role in the reproductive tract. The number of *Desulfobacteria* in the vagina of Yanbian cattle was significantly lower than that of Yanhuang cattle. Although *Desulfobacteria* can participate in catabolic reactions in the intestine by reducing sulfur compounds and degrading butyrate, etc. (Bai et al., 2022). However, the specific roles played by these two bacterial phyla in the bovine reproductive tract remain to be explored. Affected by the genetic factors of the two breeds, we found differences in bacterial genera in the reproductive tracts of the two breeds of cattle. *Delftia* was significantly higher in the vagina, cervix, and uterus of Yanbian cattle than in Yanhuang cattle; *Bacteroides* is lower than that of Yanhuang cattle. *Delftia* may serve as predictor of HPV lesion evolution (Gardella et al., 2022); *Bacteroides* metabolize polysaccharides and oligosaccharides to provide nutrients, vitamins, and other functions to the host and other intestinal microbial residents (Zafar and Saier, 2021). These results indicate that the key microbiota is significantly different in the reproductive tract of Yanbian cattle and Yanhuang cattle. The role of these species with significant differences in abundance in the reproductive tract of the two breeds remains to be explored. The relationship between these differences and the reproductive traits of Yanbian cattle and Yanhuang cattle is worth further exploration.

## Conclusion

In conclusion, our study found the commonalities and differences in the structure of the microbiota in different parts of the bovine genital tract, as well as the influence of breed factors on the composition of the bovine genital tract. These findings provide a solid theoretical basis for us to understand the reproductive health status of cattle, reveal the microecological balance of bovine reproductive tract, and guide the prevention and treatment of bovine reproductive diseases. The difference of reproductive tract microflora between Yanbian cattle and Yanhuang cattle reveals the possibility of microflora playing a role in the reproductive traits of the two breeds, which is worthy of further study.

## Data availability statement

The data that supports the findings of this study are available from the corresponding author upon reasonable request. All sequences used in this study are publicly available at the NCBI Sequence Read Archive under accession ID PRJNA1129596.

## Ethics statement

The animal studies were approved by the Ethical Committee of Jilin Agricultural University Approval Code: 20230824001. The studies were conducted in accordance with the local legislation and institutional requirements. Written informed consent was obtained from the owners for the participation of their animals in this study.

## Author contributions

YT: Formal analysis, Investigation, Software, Validation, Writing – original draft, Writing – review & editing. SF: Writing – review & editing. ZG: Formal analysis, Resources, Writing – review & editing. CH: Formal analysis, Validation, Writing – review & editing, Writing – original draft. HX: Investigation, Supervision, Writing – review & editing. ZL: Formal analysis, Writing – review & editing. JZ: Software, Supervision, Writing – review & editing. YF: Supervision, Validation, Writing – review & editing. XM: Supervision, Validation, Writing – review & editing. HL: Software, Supervision, Writing – review & editing. JG: Methodology, Supervision, Writing – review & editing. JW: Data curation, Formal analysis, Supervision, Writing – review & editing. HD: Conceptualization, Supervision, Validation, Writing – review & editing. WL: Data curation, Formal analysis, Supervision, Visualization, Writing – original draft, Writing – review & editing.
